# Development of a genotype-matched Newcastle disease DNA vaccine candidate adjuvanted with IL-28b for the control of targeted velogenic strains of Newcastle disease virus in Africa

**DOI:** 10.1007/s11259-024-10590-y

**Published:** 2024-11-25

**Authors:** Charlie F. Amoia, Augustino A. Chengula, Jean N. Hakizimana, Philemon N. Wambura, Muhammad Munir, Gerald Misinzo, James Weger-Lucarelli

**Affiliations:** 1 Department of Veterinary Microbiology, Parasitology and Biotechnology, Sokoine University of Agriculture, P. O. Box 3019, Morogoro, 67125 Tanzania; 2 SACIDS Foundation for One Health, SACIDS Africa Centre of Excellence for Infectious Diseases, Sokoine University of Agriculture, P. O. Box 3297, Morogoro, 67125 Tanzania; 3 Center for Emerging, Zoonotic, and Arthropod-borne Pathogens, Virginia Polytechnic Institute and State University (Virginia Tech), Blacksburg, VA 24061 USA; 4 OR Tambo Africa Research Chair for Viral Epidemics, SACIDS Foundation for One Health, Sokoine University of Agriculture, P. O. Box 3297, Morogoro, 67125 Tanzania; 5 Division of Biomedical and Life Sciences, Faculty of Health and Medicine, Lancaster University, Lancaster, LA1 4YG UK; 6 Department of Biomedical Sciences and Pathobiology, Virginia-Maryland College of Veterinary Medicine, Virginia Tech, Blacksburg, VA 24060 USA

**Keywords:** Newcastle Disease virus, Genotype-matched, DNA vaccine, Interferon lambda-3, IL-28b, Molecular adjuvant

## Abstract

Newcastle disease virus (NDV) is an extremely contagious and deadly virus that affects numerous bird species, posing serious threats to poultry production on a global scale. In addition to implementing biosecurity practices in farming systems, vaccination remains the most effective means of controlling Newcastle disease (ND). However, while existing commercial vaccines provide some level of protection, the effectiveness of these vaccines can be questionable, particularly in field settings where the complexity of vaccination program implementation poses significant challenges, especially against virulent genotypes of NDV. A genotype-matched NDV DNA vaccine could potentially offer a more effective vaccination approach than currently available live attenuated vaccines. By being specifically tailored to match circulating strains, such a vaccine might improve efficacy and reduce the risk of vaccine failure due to genotype mismatch. To develop an alternative vaccine approach, two ND DNA vaccines were constructed in this study. Each vaccine developed in this study contains the fusion (F) and haemagglutinin-neuraminidase (HN) genes of a virulent NDV genotype VII isolate from Tanzania. Interferon lambda-3 (IFNλ3; IL-28b), which has demonstrated capacity to significantly enhance specific adaptive immune responses and decreased levels of inflammatory cytokines, as well as improved protective responses at a high viral challenge dose, was included in one of the developed vaccines. These plasmids were designated pTwist-F-HN-VII-IL28b and pTwist-F-HN-VII. The two plasmids differed in that pTwist-F-HN-VII-IL28b contained the cytokine adjuvant IL-28b. Transfection of cells and subsequent immunofluorescence assays indicated that both plasmids expressed high levels of NDV F-HN proteins. In vivo immunization demonstrated that chicks intramuscularly immunized with pTwist-F-HN-VII-IL28b exhibited significant immune responses compared to chicks immunized with pTwist-F-HN-VII or the commonly used LaSota vaccine (LaSota), which was used as a control. The protective efficacy of pTwist-F-HN-VII-IL28b was 80% after challenge with the highly virulent NDV strain ON148423, compared to 60% for chicks vaccinated using LaSota, and pTwist-F-HN-VII. The findings of this study indicate that IL-28b can be employed as a molecular adjuvant for NDV vaccines. This study represents a key milestone in Newcastle disease vaccine research, particularly in the development of a genotype-matched DNA vaccine candidate. Additionally, this study demonstrated that the combination of F, HN, and IL-28b elicits an efficacious immune response against virulent NDV strains.

## Introduction

Based on 2021 data from the Tanzanian National Bureau of Statistics (NBS), over 80% of Tanzanian households raise chickens, playing a crucial role in the agricultural GDP. Indigenous chickens account for more than 70% of the chicken meat and egg demand in rural regions and up to 20% in urban areas. Nationwide, there are roughly 40 million indigenous chickens, while commercial poultry numbers around 32 million, with 24 million raised for meat and 8 million for egg production (CSIRO 2020). All this poultry production and its importance to the country’s food security have one main threat, Newcastle disease (ND).

Newcastle disease is a serious disease of poultry that can cause severe economic losses in many countries (De Leeuw and Peeters 1999). While there is currently no evidence of human-to-human transmission, transmission can occur from birds to humans, usually causing mild disease. However, in rare cases, it may pose a more severe risk, particularly to immunocompromised individuals. Thus, NDV is a potential zoonotic threat. Vaccination is the most effective way to control ND (Dimitrov et al. 2017). This disease is enzootic in many parts of the world and has a significant worldwide influence on the poultry industry (Saad et al. 2017; Umar 2017; Hassanzadeh et al. 2024). *Avian orthoavulavirus 1* (AOaV-1), previously known as Newcastle disease virus (NDV), the causative agent of ND, belongs to the family *Paramyxoviridae*, subfamily *Avulavirinae*, genus *Orthoavulavirus* (Walker et al. 2019). The transmission of NDV occurs primarily via airborne contact, direct contact with respiratory discharge or feces from infected birds, or indirect contact with contaminated feed, water, litter, equipment, or other objects (Brown and Bevins 2017).

Newcastle Disease Virus (NDV) is characterized by its enveloped form and a non-segmented, single-stranded, negative-sense RNA genome. This genome comprises six genes, which are ordered from the 5′ to the 3′ end in the sequence of nucleocapsid protein (N), phosphoprotein (P), matrix protein (M), fusion protein (F), hemagglutinin-neuraminidase protein (HN), and large polymerase protein (L). Two additional proteins, V and W, are expressed by RNA editing of P messenger RNA (mRNA) (Lamb and Parks 2007). The F and HN proteins of NDV are critical for its infectivity and immunogenicity, making them essential components of effective vaccines (Ge et al. 2016; Lu et al. 2024). The F protein facilitates the fusion of the viral envelope with the host plasma membrane, allowing the virus to enter and infect the host cell (Taghizadeh et al. 2024). Together, these proteins elicit strong immune responses, producing neutralizing antibodies that prevent the virus from infecting cells and spreading (Smith et al. 2023). The HN protein is responsible for binding to host cell surface receptors and plays a role in the release of new virions from infected cells (Yang et al. 2024). Vaccines that include F and HN proteins provide robust protection against NDV by targeting viral entry and spread (Liu 2015). The control of ND is possible through the use of vaccines (Mayers et al. 2017; Lindahl et al. 2019; Zenglei et al. 2022). NDV is highly diverse, with 21 genotypes identified to date (Dimitrov et al. 2019). While existing live-attenuated commercial vaccines are effective in preventing mortality, they often require intensive vaccination programs and may not fully prevent viral shedding or cross-protect against other virulent genotypes (Sultan et al. 2020; Bello et al. 2020; Ferreira et al. 2021). Previous studies have demonstrated that genotype-matched vaccines are more effective than genotype II-based vaccines (Izquierdo-Lara et al. 2019a; Bello et al. 2020; Sultan et al. 2022). In particular, NDV strains of genotype VII are the main NDV genotypes identified in Tanzania (da Silva et al. 2020; Amoia et al. 2023; Kariithi et al. 2023). Several studies in different parts of the world have reported that genotype VII is responsible for many of the current NDV outbreaks in poultry in Africa and Asia (Snoeck et al. 2009; Dimitrov et al. 2016; Ewies et al. 2017). Importantly, genotype VII is highly virulent, causing significant losses in the poultry industry.

ND outbreaks have persisted in vaccinated poultry, suggesting that both the genetic variations between vaccine strains and field viruses, as well as potential issues with vaccine administration, play critical roles in the ineffectiveness of current vaccines (Umali et al. 2013; Zenglei et al. 2022). Thus, the inactivated and attenuated live vaccines currently in use against ND have limitations, including their effectiveness against virulent strains of NDV, safety concerns due to the possibility of reversion, and cold chain requirements, especially for developing countries where access to electricity in rural areas is still very low (Milic et al. 2017; Shahar et al. 2018; Bello et al. 2020). Several vaccines, including live, inactivated, subunit, and recombinant vaccines, have been continuously explored for more effective prophylactic measures, but none of them meet all the desired criteria (Dimitrov et al. 2017).

The continuous evolution and emergence of new NDV genotypes present ongoing challenges for ND control, especially in Africa, where the virus exhibits considerable genetic diversity. Findings from recent studies on NDV genotypes across Africa in general and, more specifically, in Tanzania emphasize the need for improved diagnostic and vaccine strategies (Amoia et al. 2024a). DNA vaccines offer several advantages for preventing ND in poultry. One of the key benefits of DNA vaccines is their capacity to induce robust and sustained cellular and humoral immune responses (Lu et al. 2024).

In contrast to traditional inactivated and live-attenuated vaccines, DNA vaccines offer the advantage of not posing a risk of reverting to virulence or inducing the disease they are designed to prevent, providing an enhanced safety profile for both poultry and humans handling the vaccines (Zhao et al. 2014; Kim and Samal 2016). In addition, DNA vaccines represent a promising technology for developing countries due to their safety, genetic stability, ease of production, lack of cold chain requirements, and activation of innate immune pathways (Lu et al. 2008).

A number of studies have been conducted on the construction of ND DNA vaccines. These vaccines have sometimes been adjuvanted with different chicken interferons and/or encapsulated in nanoparticles (Zhao et al. 2013, 2017; Mohebbi et al. 2019; Xie et al. 2020). Furthermore, it has been demonstrated that adding a cytokine adjuvant to a DNA vaccine can enhance immunogenicity (Ciabattini et al. 2016). In particular, the use of interferon lambda-3 (IFNλ3; IL-28b) as an adjuvant has been demonstrated to significantly enhance specific adaptive immune responses and decrease the levels of inflammatory cytokines, as well as improve protective responses at a high viral challenge dose for influenza (Sabbaghi et al. 2021; Wallace et al. 2021; Mallampalli et al. 2021). In this respect, an ND DNA vaccine expressing the F and HN proteins of genotype VII and adjuvanted with IL-28b could offer an alternative solution for a better fight against ND. Employing a vaccine strain identical to the prevalent viruses is expected to lower viral shedding and thus prevent transmission between vaccinated chickens (Miller et al. 2013). Genotype-matched vaccines have been shown to induce a more robust and specific immune response compared to non-genotype-matched vaccines, particularly when used in challenge studies with virulent strains that correspond to the vaccine’s genotype (Izquierdo-Lara et al. 2019b; Sultan et al. 2022). In these studies, vaccinated animals were challenged with homologous virulent NDV strains, and parameters such as immune response, morbidity, and mortality were evaluated. The results consistently demonstrated that genotype-matched vaccines provide enhanced protection, reduce virus shedding, and improve survival rates, underscoring their potential benefits over traditional vaccines that may not fully protect against diverse NDV genotypes. The objective of this study was to develop a genotype-matched NDV DNA vaccine adjuvanted with IL-28b using the F and HN genes of an NDV strain of genotype VII isolated from Tanzania. In addition, the protective immune response induced by the developed genotype-matched NDV DNA vaccine candidate was evaluated through an in vivo experiment in chicks.

## Materials and methods

### Cells

BHK-21 clone 13 (baby hamster kidney fibroblasts, ATCC^®^ CCL-10™) cells were maintained at 37 °C in a 5% CO_2_ humidified incubator using Dulbecco’s modified Eagle’s medium (DMEM; Thermo Fisher Scientific, Carlsbad, CA, USA) supplemented with 5% fetal bovine serum (FBS), 1% nonessential amino acids, 0.1% gentamicin and 25 mM HEPES buffer (herein the cell culture medium is referred to as DMEM-5) (Rai et al. 2021). BHK-21 cells were used based on their high transfection efficiency (Yin and Kielian 2021).

### Viruses

The sequences of two velogenic strains of NDV collected in Tanzania’s Morogoro and Iringa regions were used to design the ND DNA vaccines. The F gene sequence (chicken/Tanzania/Iri1921/2022, PP216548) of the strain isolated from Iringa and the HN gene sequence (chicken/Tanzania/Mor1221/2022, PP195786) of the strain isolated from Morogoro have been used in the construction of the vaccine (Amoia et al. 2024b). The NDV strain (chicken/Tanzania/Sum4/2021, accession number ON148423) (Amoia et al. 2023), isolated in 2021 from an outbreak of ND in chickens in Tanzania and identified as velogenic, was used for the challenge study.

### Plasmid construction

Sequences for the F gene (accession number PP216548) and the HN gene (accession number PP195786) were obtained by Sanger sequencing. The sequence of chicken IL28b was derived from GenBank (EU399904). The T2A sequence, derived from Thosea asigna virus (Luke et al. 2008; Birnbaum et al. 2017), and the P2A sequence, derived from porcine teschovirus-1 (Sladitschek and Neveu 2015; Luke and Ryan 2018), were used. The Gly-Ser-Gly (GSG) linker was copied from the 2 A peptide-linked multicistronic vector designed and constructed by Szymczak-Workman et al. (2012). The Kozak sequence selected was the conserved vertebrate Kozak sequence (Nakagawa et al. 2008).

**Fig. 1:**
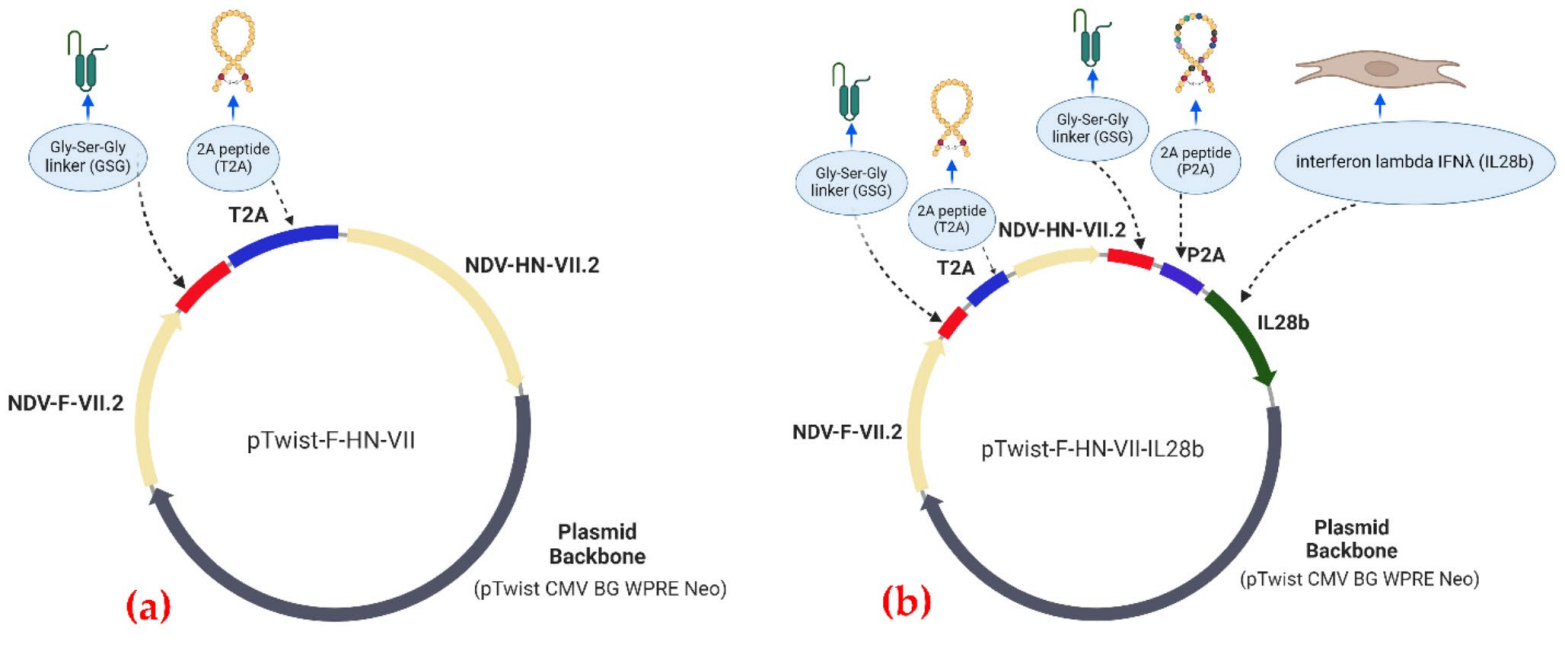
The first vaccine cassette was designed as follows: Kozak sequence - F gene without the stop codon– GSG linker - T2A - HN gene without the stop codon - GSG linker -P2A. This cassette was then cloned and inserted into a cytomegalovirus (CMV) driven expression vector, named pTwist CMV WPRE Neo, which contains a beta-globin intron and a woodchuck hepatitis virus post-transcriptional regulatory element (WPRE) with *EcoR*I and *Xba*I insertions (Twist Bioscience, South San Francisco, CA, USA). The second vaccine cassette was designed as follows: Kozak sequence - F gene without the stop codon– GSG linker - T2A - HN gene without the stop codon - GSG linker -P2A– IL-28b. This cassette was also cloned and inserted into the CMV-driven expression vector pTwist CMV WPRE Neo

Two vaccines were developed for this study. The first vaccine, pTwist-F-HN-VII, was developed using only the F and HN genes of the NDV subgenotype VII.2 isolated from Morogoro and Iringa in Tanzania (Fig. 1a). The second vaccine, pTwist-F-HN-VII-IL28b, contained the HN and F genes of the NDV subgenotype VII.2 as in the first vaccine, along with IL-28b as an adjuvant (Fig. 1b).

### Plasmids generic stock preparation

After receiving the plasmids (pTwist-F-HN-VII-IL28b and pTwist-F-HN-VII) from Twist Bioscience, a high-concentration plasmid stock was prepared for downstream use. After transformation into NEBStable competent cells by electroporation (Kisakov et al. 2024), the plasmids were incubated for 18 h at 30 °C. Colonies were picked and incubated in terrific broth (TB) media supplemented with carbenicillin at a concentration of 100 µg/mL. DNA was extracted using a ZymoPure miniprep kit, and subsequently, the ZymoPURE II Plasmid Midiprep and Gigaprep Kits (Zymo Research, Irvine, CA, USA) were used to produce high-concentration plasmid stocks for restriction digestion, transfection, indirect immunofluorescence assay (IFA), and vaccination tests in chicks. To increase our plasmid yield, we added a subinhibitory concentration of 3 µg/mL chloramphenicol to the cultures prepared for Midiprep and Gigaprep for further incubation for 12 h at 30 °C and 200 rpm. The culture was then incubated overnight before plasmid purification. Plasmid quality and concentration were determined using a Nanodrop (Thermo Fisher Scientific, Waltham, MA, USA) and Invitrogen’s Qubit 1x dsDNA HS kit (Thermo Fisher Scientific, Carlsbad, CA, USA). Restriction digestion and gel electrophoresis were used to verify the correct banding and size of the plasmid DNA.

### Transfection and indirect immunofluorescence assay

To introduce the ND DNA plasmids (pTwist-F-HN-VII and pTwist-F-HN-VII-IL28b) into cells, and observe the expression of the F and HN genes within the cells, transfection process was used. BHK-21 cells were seeded at a density of 10^4^ cells/mL per well in a 96-well flat-bottom plate containing DMEM-5. On day 2, ND DNA plasmids (pTwist-F-HN-VII and pTwist-F-HN-VII-IL28b) were transfected into the cells to assess expression using jetOPTIMUS DNA transfection reagent (Polyplus Transfection, Illkirch, France), according to the manufacturer’s instruction, with a DNA concentration of 0.13 µg per well in a 96-well flat-bottom plate. For the infection of BHK-21 cells with the NDV LaSota strain (live-attenuated vaccine), a multiplicity of infection (MOI) of 4 was used, and the infection was carried out for 48 h in the same 96-well plates.

To confirm the expression of F and HN proteins from the two plasmids, in BHK-21 cells, indirect immunofluorescence assay was used.Thus, on day 3, the transfected cells were stained using immunofluorescence assay (IFA) following a standardized protocol (Chumbe et al. 2017). Cells were rinsed with phosphate-buffered saline (PBS) and fixed with 10% formalin for 20 min at room temperature (RT). After formalin removal, cells were washed thrice with PBS-Tween (PBS-T) (PBS with 0.1% Tween 20), followed by incubation with permeabilization buffer (0.1% Triton X-100 in 1x PBS) for 10 min at RT. Cells were washed again with PBS-T and blocked with blocking buffer (1% BSA, 22.52 mg/mL glycine in PBS-T) for 30 min at RT. Following additional washing steps with PBS-T, cells were incubated with a primary antibody, polyclonal chicken anti-Newcastle disease virus antibody (ab34402, Abcam), diluted 1:800 in 1% BSA in PBS, for 1 h at RT. After washing, the cells were incubated with an Alexa Fluor 488-conjugated anti-chicken IgG antibody, at a dilution of 1:400, in the dark for 1 h at RT. Further washing was performed before staining the nuclei with 1 µg/mL DAPI in PBS for 1 min, followed by a final rinse with PBS. Visualization of cell staining was achieved by adding 100 µL of PBS into the wells.

### Experimental design for the test of vaccine efficacy

As part of the in vivo study of our two new ND DNA vaccines (pTwist-F-HN-VII and pTwist-F-HN-VII-IL28b), a pilot study was carried out on 80 chicks (broilers) (Interchick Company Ltd, Dar es Salaam, Tanzania) to assess the immunogenicity and efficacy of our vaccines and select the best vaccine administration route for use in phase II of our experiment. The day-old chicks used in this in vivo study were purchased from a commercial hatchery in Dar es Salaam, Tanzania. The experimental birds were kept in poultry houses at the experimental station of the Department of Animal, Aquaculture and Range Sciences, Sokoine University of Agriculture, Morogoro, Tanzania, under controlled conditions for the duration of the study and received regular feed and water *ad libitum*.

#### Newcastle disease DNA vaccines vaccination pilot study experiment I

**Fig. 2:**
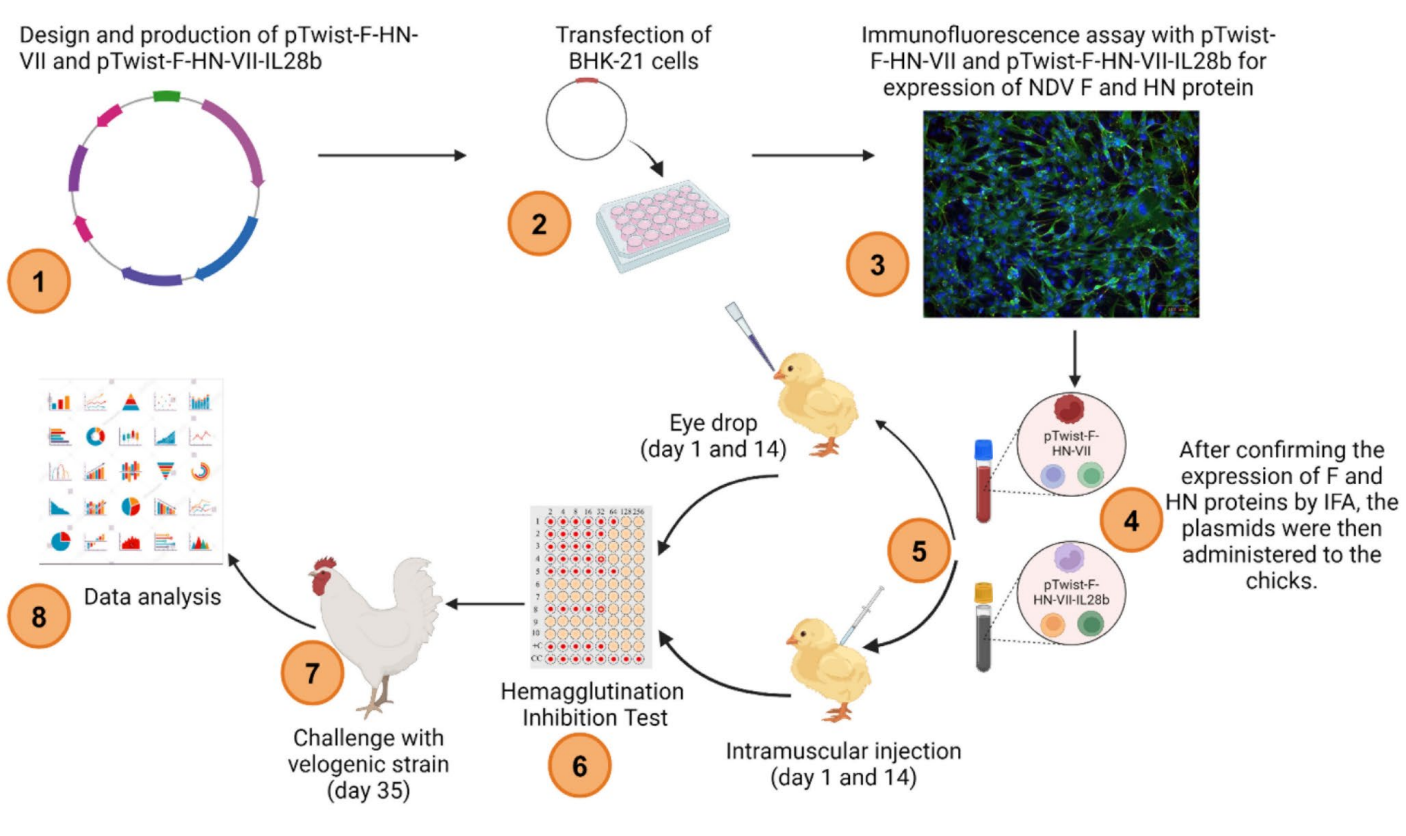
Presentation of the pilot study using the candidates’ vaccines as an example

For the pilot study (Fig. 2), a total of 80 one-day-old chicks (broilers) were divided into 4 experimental groups with 4 vaccination protocols (Table 1). Each chick in the group vaccinated with pTwist-F-HN-VII-IL28b or pTwist-F-HN-VII received 100 µg of plasmid DNA on day one and day fourteen via intramuscular (IM) injection or by eye drop. The dose was administered in a total volume of 100 µL per chick to ensure adequate delivery either intramuscularly or via eye drop. On days 7, 14, 21, 28, and 35 after immunization, blood samples were taken aseptically from each chicken’s brachial vein. The serum was extracted by allowing the blood to coagulate and centrifuge before the serum was transferred to a new tube. After that, the serum samples were frozen at -20 °C until their levels of NDV-specific antibodies were measured using the hemagglutination inhibition (HI) test. The maternal antibody levels against NDV were assessed for all sets of animal trials using the HI test.

**Table 1:** Comparison of various routes of administration of pTwist-F-HN-VII, pTwist-F-HN-VII-IL28b, and LaSota at 1 day of age

Vaccine	Number of chicks per group	Route of administration	Vaccination Schedule	
			Prime Vaccination (Day 1)	Boost Vaccination (Day 14)
pTwist-F-HN-VII	10	Intramuscular injection	++	++
	10	Eye Drop	++	++
pTwist-F-HN-VII-IL28b	10	Intramuscular injection	++	++
	10	Eye Drop	++	++
LaSota	10	Intramuscular injection	++	++
	10	Eye Drop	++	++
None (unvaccinated controls)	20	None	--	--

*++: Yes

*--: No

#### Newcastle disease DNA vaccines vaccination study experiment II

**Fig. 3:**
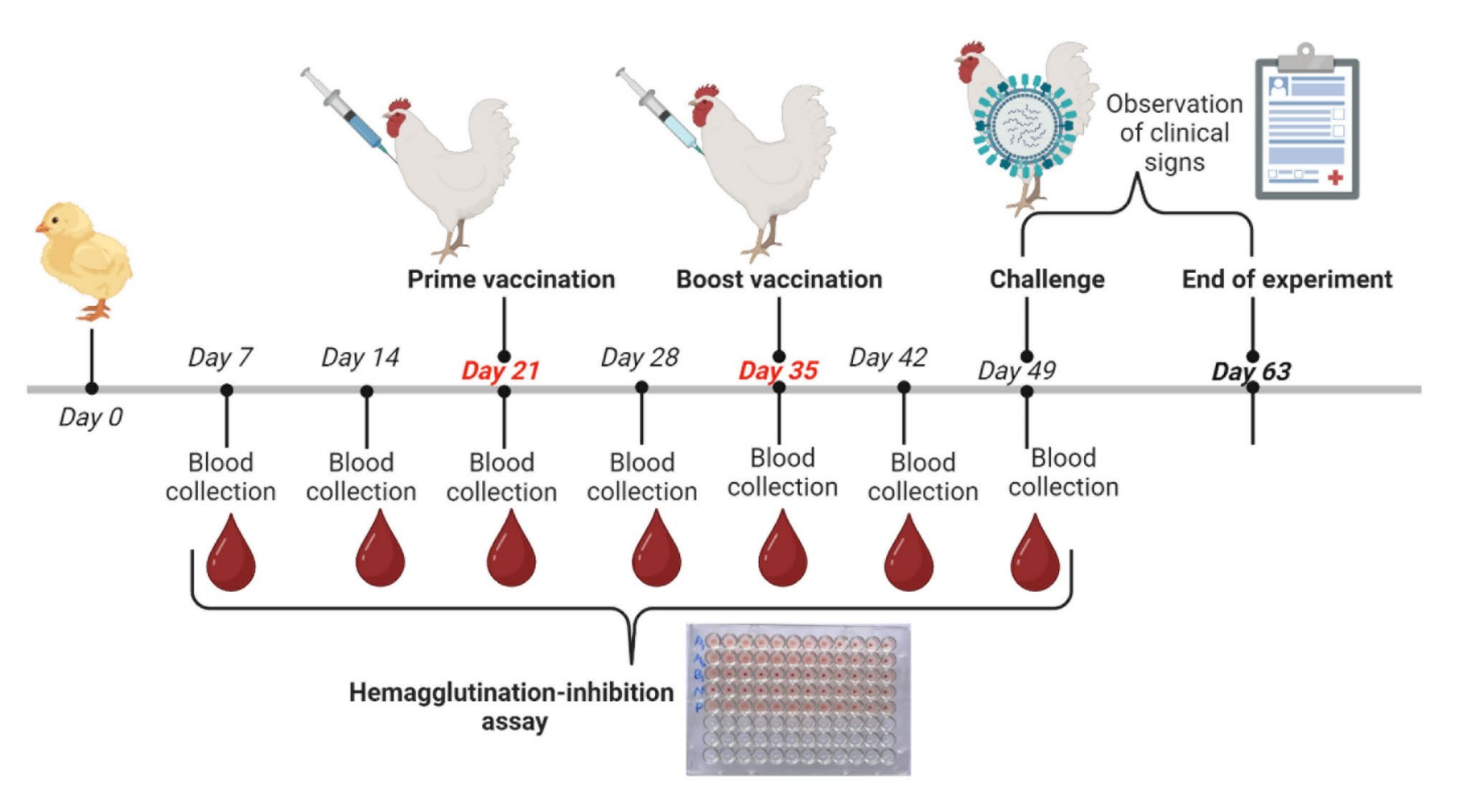
Immunization schedule and blood sampling intervals in chicks during the phase II vaccination experiment

For the phase II experiment (Fig. 3), 120 one-day-old broiler chicks were divided into experimental groups with 5 vaccination protocols (Table 2). All chicks were identified with numbered tags. Each chick in the group vaccinated with pTwist-F-HN-VII-IL28b or pTwist-F-HN-VII received 100 µg of plasmid DNA on day 21 and on day 35 via intramuscular (IM) injection. The dose was administered in a total volume of 100 µL per chick. A 3 mL sterile disposable syringe was used to collect 1-1.5 mL of blood per bird from the wing vein at days 7 and 14 before vaccination and then at days 21, 28, 35, 42 and 49. The NDV-specific antibody titers in the collected blood samples were then determined using the HI test. The maternal antibody levels against NDV were assessed for all sets of animal trials using the HI test.

**Table 2:** Phase II vaccination protocols

Vaccine	Number of chicks per group	Route of administration	Vaccination Schedule	
			Prime Vaccination (Day 21)	Boost Vaccination (Day 35)
pTwist-F-HN-VII	30	Intramuscular injection	++	++
pTwist-F-HN-VII-IL28b	30	Intramuscular injection	++	++
LaSota	30	Intramuscular injection	++	++
None (unvaccinated controls)	30	None	--	--

*++: Yes

*--: No

#### Randomization before challenge

The challenge facility was an isolated bio-secure facility at the Department of Animal, Aquaculture and Range Sciences of SUA. Chickens had *ad libitum* access to feed and water. To enable the challenge phase to be carried out in such a way that all challenged animals, regardless of the group to which they belong, have the same probability of death or survival following challenge with NDV velogenic genotype VII, a randomized selection process was used. Microsoft Excel was used to randomly select 5 chickens in each group for the challenge phase. To ensure that the animals in the different treatment groups were exposed to the virus in the same way throughout the challenge step, they were housed in the same room, fed the same feed and watered with the same water during the post-challenge period. The virus embryo infective dose 50 (EID_50_) was administered to each animal (Reed and Muench 1938a) by a technician from SUA’s Virology Laboratory, who was not involved in our study and had no idea of the treatment group to which each animal to be infected belonged.

#### Newcastle disease challenge study

In the challenge study, four groups of chickens were used: (1) chickens vaccinated with pTwist-F-HN-VII, (2) chickens vaccinated with pTwist-F-HN-VII-IL28b, (3) chickens vaccinated with the LaSota strain, and (4) an unvaccinated control group. All groups, including the unvaccinated controls, were challenged with a virulent strain of Newcastle Disease Virus (NDV). The unvaccinated group served as the control to evaluate the baseline susceptibility to infection and to compare the protective effects of the different vaccines. The NDV strain (ON148423) used for the challenge study was identified as velogenic and classified as subgenotype VII.2 through phylogenetic analysis (Amoia et al. 2023). The virus was propagated in embryonated chicken eggs that were 10 days old. To determine the virus EID_50_, titration was performed following the procedure described by Reed and Muench (1938b). The challenge virus was administered intranasally. Following the challenge, daily observations of the birds were conducted to check for death and symptoms of illness, such as oedema, muscle tremors, torticollis, and paralysis of the wings and legs. The birds were observed twice a day.

### Statistical analysis

All serological data are expressed as the mean values ± standard deviations (SD). The data were subjected to one-way ANOVA and multiple comparisons in R-4.4.0.0 software to evaluate the significant differences among different groups. Differences between groups with *p* < 0.05 were considered to be statistically significant.

## Results

### Transfection and in vitro expression of F and HN glycoproteins

**Fig. 4:**
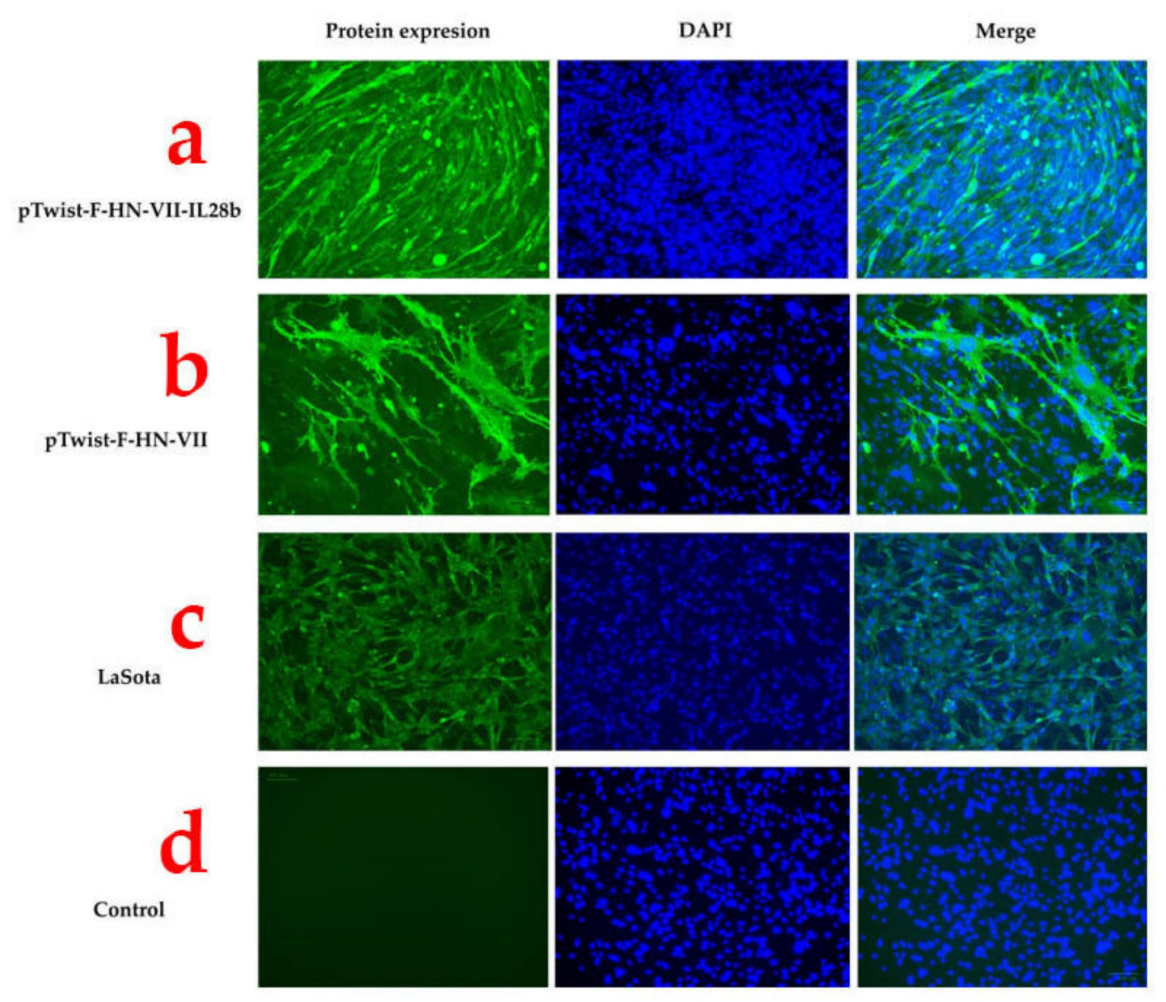
Expression of the envelope F and HN glycoproteins was observed in the transfected cells. BHK cells that had been transfected were treated with a primary anti-NDV polyclonal antibody specific to the Newcastle disease virus (ab34402, Abcam) and subsequently with an anti-chicken IgG antibody. Cells transfected with the negative control (PBS) showed no reaction to the primary antibody (d)

To confirm the expression of NDV proteins from the two plasmids, we used an indirect immunofluorescence assay. The cells were transfected and fixed 48 h later. The cells were treated with a polyclonal anti-Newcastle disease virus antibody and an Alexa Fluor 488-conjugated secondary antibody. DAPI was used to stain the nuclei for visualization purposes. We observed high levels of expression for both plasmids (Fig. 4a-b) as well as for the control wells infected with the LaSota vaccine strain (Fig. 4c). We observed no signal in the mock-treated wells (Fig. 4d). In conclusion, we generated an ND genotype-matched DNA vaccine that express either F and HN proteins.

### Determination of route of immunization

To compare inoculation routes of the two plasmid vaccine strategies, a pilot study was first performed. Table 3 illustrates the antibody titers obtained during the pilot phase after vaccination of the chicks. Unvaccinated chickens in the negative control group demonstrated no evidence of an anti-NDV immune response. In contrast, the other groups of chickens vaccinated with the positive control (LaSota) and the two ND DNA candidate vaccines exhibited antibody titers that increased over time, peaking one week after booster injection (Table 3). The statistical analysis of the pilot study results revealed a significant difference between the two routes of administration (*P* < 0.05). The results observed with IM-vaccinated chicks demonstrated significant antibody responses across all vaccine groups. This outcome prompted the selection of the intramuscular (IM) route as the administration method during the second phase of vaccination.

**Table 3:** Hemagglutination Inhibition Titer in the Vaccinated and Nonvaccinated groups for Experiment I

	Vaccination	Control	LaSota		pTwist-F-HN-VII-IL28b		pTwist-F-HN-VII	
		Unvaccinated	Eye drop	IM	Eye drop	IM	Eye drop	IM
Day 0	First	NA	NA	NA	NA	NA	NA	NA
Day 7		1.5 ± 0.17^n^	2.4 ± 0.16^m^	2.8 ± 0.25^lm^	3.3 ± 0.15^kl^	4.5 ± 0.17^i^	3 ± 0.21^l^	2.8 ± 0.25^lm^
Day 14	Booster	0.8 ± 0.2^o^	3.1 ± 0.18^l^	4.4 ± 0.22^i^	4.4 ± 0.16^i^	5.4 ± 0.16^gh^	4.2 ± 0.13^ij^	4 ± 0.26^ij^
Day 21		0.4 ± 0.16^op^	3.7 ± 0.21^jk^	5.2 ± 0.2^h^	5.8 ± 0.13^fg^	7.1 ± 0.18^cd^	5.4 ± 0.16^gh^	5.4 ± 0.34^gh^
Day 28		0.2 ± 0.13^p^	4.4 ± 0.16^i^	5.7 ± 0.26^gh^	7.4 ± 0.27^bc^	8.6 ± 0.16^a^	6.3 ± 0.15^ef^	6.6 ± 0.31^de^
Day 35		0.1 ± 0.1^p^	4.5 ± 0.22^i^	5.9 ± 0.18^fg^	7.7 ± 0.21^b^	9.1 ± 0.23^a^	6.8 ± 0.13^de^	7.4 ± 0.22^bc^

*Values are presented as the mean ± standard deviation of seven experiments in each group; values within the same column with different superscripted lowercase letters (a–p) are significantly different (p < 0.05) due to sampling-day effects and treatment effects. The significance between groups was analyzed using repeated measures ANOVA followed by a post hoc test (Tukey’s HSD), with comparisons made at each time point

*NA= No blood collection for chicks on this date

### Results of immunization in experiment II

**Fig. 5:**
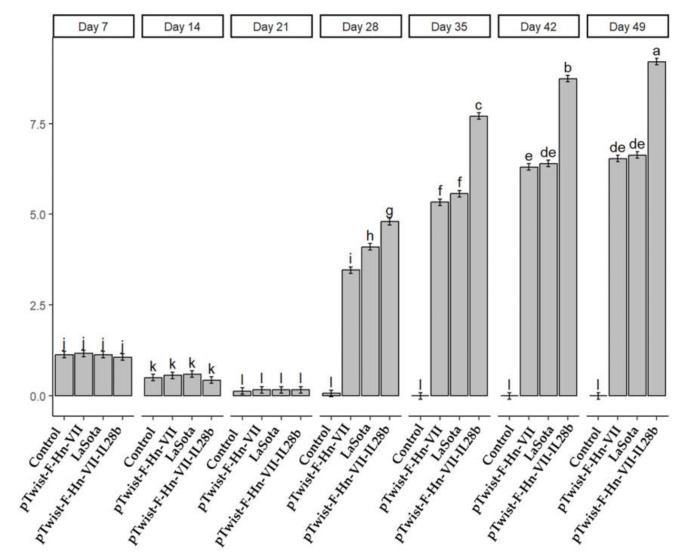
Dynamics of HI titers of NDV-specific antibodies (mean log2) during experiment II after immunization of chicks with LaSota (eye drop and IM), plasmid pTwist-F-HN-VII (eye drop and IM) and pTwist-F-HN-VII-IL28 (eye drop and IM). ANOVA followed by a post hoc test was performed to determine which groups (time points or vaccines) significantly differed. A side-by-side comparison of Mean LogHI values across different time points for each vaccine was performed. Each facet shows the performance of pTwist-F-HN-VII-IL28b compared with that of LaSota, pTwist-F-HN-VII, and negative control regarding Mean LogHI values over time. Values within the same column with different superscripted lowercase letters (a–p) are significantly different (*p* < 0.05) due to sampling-day effects and treatment effects

A comparison of the route of inoculation of our two plasmid vaccines and that of LaSota during the pilot study revealed that intramuscular injection resulted in significant outcomes. This route of administration was subsequently employed for the administration of the various vaccines to a larger number of animals. Table 4 illustrates the antibody titers obtained during the pilot phase, before and after vaccination of the chicks. Unvaccinated chickens in the negative control group demonstrated no evidence of an anti-NDV immune response (Fig. 5). One week following the initial injection, the antibody titers of chickens immunized with pTwist-F-HN-VII-IL28b were significantly greater than those of the other vaccinated groups. In the third week following the first immunization, the titers peaked and remained elevated for an extended period. These findings indicated that pTwist-F-HN-VII-IL28b induced more robust immune responses than did pTwist-F-HN-VII and LaSota vaccines. This disparity was further accentuated following booster injection in chickens that had been immunized with pTwist-F-HN-VII-IL28b.

**Table 4:** Hemagglutination inhibition titer in vaccinated and nonvaccinated groups for experiment II using only IM injection

Time	Vaccination	Control	LaSota	pTwist-F-HN-VII-IL28b	pTwist-F-HN-VII
Day 7		1.13 ± 0.06^j^	1.13 ± 0.06^j^	1.07 ± 0.05^j^	1.17 ± 0.07^j^
Day 14		0.5 ± 0.09^k^	0.6 ± 0.09^k^	0.43 ± 0.09^k^	0.57 ± 0.09^k^
Day 21	First	0.13 ± 0.06^l^	0.17 ± 0.07^l^	0.17 ± 0.07^l^	0.17 ± 0.07^l^
Day 28		0.07 ± 0.05^l^	4.1 ± 0.11^i^	4.8 ± 0.12^h^	3.47 ± 0.12^g^
Day 35	Booster	0.0 ± 0.0^l^	5.57 ± 0.11^f^	7.7 ± 0.15^f^	5.33 ± 0.12^c^
Day 42		0 0.0 ± 0.0^l^	6.4 ± 0.09^e^	8.73 ± 0.11^de^	6.3 ± 0.12^b^
Day 49		0.0 ± 0.0^l^	6.63 ± 0.1^de^	9.2 ± 0.1^de^	6.53 ± 0.09^a^

*Values are presented as the mean ± standard deviation of seven experiments in each group; values within the same column with different superscripted lowercase letters (a–p) are significantly different (p < 0.05) due to sampling-day effects and treatment effects

### Results post-challenge

**Fig. 6:**
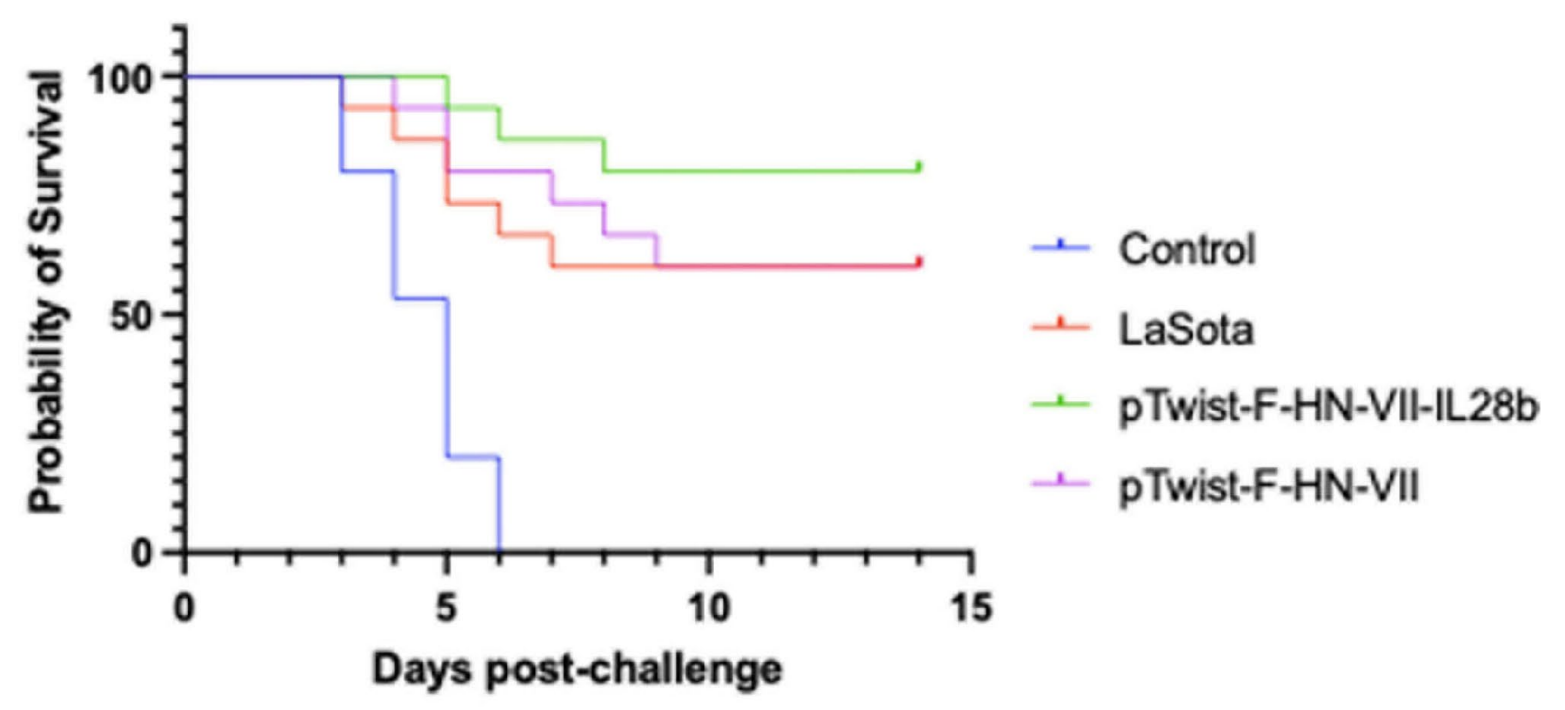
Survival rate curves of the vaccinated and unvaccinated groups after challenge were generated via Kaplan‒Meier curves and log-rank (Mantel–Cox) tests

In the two immunization schemes conducted in this study, the various groups of chickens were challenged with the velogenic NDV virus two weeks after the booster. Five chickens were challenged for each group in the pilot phase, and 15 chickens were challenged in the second immunization scheme (Fig. 6). LaSota and pTwist-F-HN-VII were shown to provide a limited amount of protection (60%), as shown in Tables 3 and 4. However, a significant level of protection was observed with the adjuvanted vaccine (pTwist-F-HN-VII-IL28b) obtained with the initial immunization schedule. In both immunization schemes, the chickens in the control group (unvaccinated) succumbed to infection with the velogenic NDV virus within 5 to 6 days. Chickens in the control group (unvaccinated) showed signs of depression, nasal discharge, respiratory distress, and bright green diarrhea at 3 to 4 days postchallenge (dpc). Chickens in the LaSota group (in the two immunization schemes) showed mild clinical symptoms of lassitude, rejected yellow and green feces and had watery eyes beginning on the second day after challenge, and 60% of the chickens survived to the end of the study at 14 dpc. The chickens that were immunized with pTwist-F-HN-VII and exposed to the virulent strain showed mild clinical symptoms of lassitude, rejected yellow and green feces and had watery eyes from the second day after challenge, and 60% of the chickens survived to the end of the study. The chickens that were immunized with pTwist-F-HN-VII-IL28b and exposed to the virulent strain exhibited normal feeding, watering, and mental state behaviors. However, some chickens exhibited mild respiratory distress, sneezing, and coughing at 8 dpc, and 80% of the chickens survived to the end of the study. The Cox model for the hazard of mortality (risk of death) is influenced by the variables “Normal” and “Sick” (Status of chicken). The coefficient for “Normal” was − 0.10989, with a corresponding hazard ratio of 0.89593, suggesting a slight decrease in the hazard of death, although the difference was not significant at the α = 0.05 level (*p* = 0.0585). Conversely, the coefficient for “Sick” was 0.27310, indicating an increase in the hazard of death, with a hazard ratio of 1.31403, which was statistically significant (*p* = 0.0070). This finding implies that being sick significantly increases the mortality risk among vaccinated chickens.

### Necropsy outcomes following challenge

**Fig. 7:**
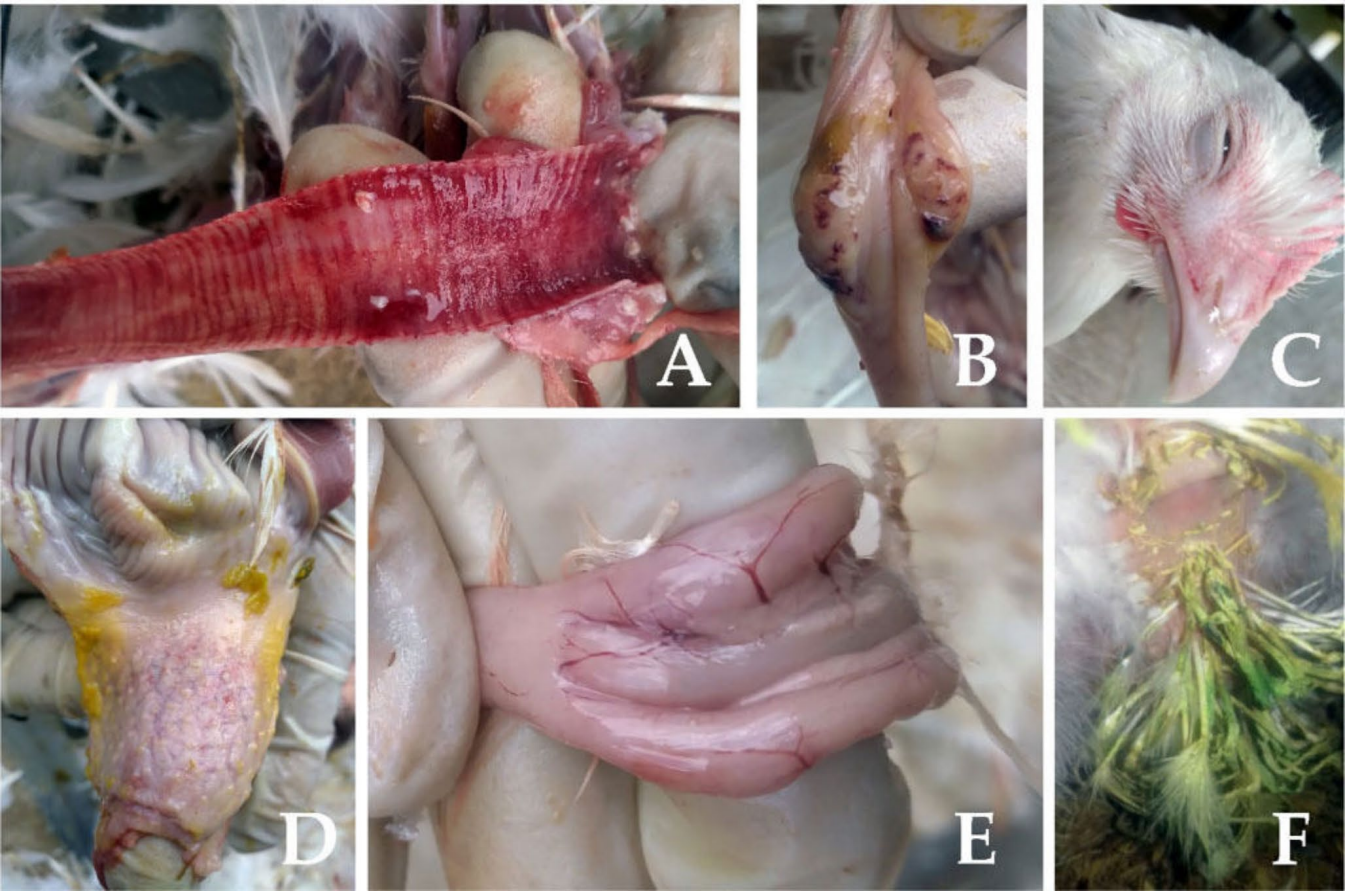
Results of clinical observation of some vaccinated and unvaccinated birds after field challenge with the virulent NDV strain. (**A**) Hemorrhage in the mucosa of the trachea. (**B**) Marked acute hemorrhage and edema in the cecal tonsils. (**C**) Edema and swelling of the eyelid. (**D**) Multifocal hemorrhages in the mucosa of the proventriculus. (**E**) Necrosis of lymphoid tissue at the cecal tonsils. (**F**) Green watery diarrhea

Upon necropsy, the majority of the deceased birds exhibited pathological lesions that were characteristic of ND (Fig. 7), including hemorrhages in the tracheal mucosa, petechiae in the mucosa of the proventriculus, and marked acute hemorrhages and edema in the cecal tonsils.

## Discussion

Although all African countries currently implement vaccination programs against ND, a considerable number of outbreaks are still reported on the continent on an annual basis. This situation may indicate that the vaccines and/or vaccination practices currently used in Africa are ineffective at controlling ND, as has been the case in other parts of the world (Mayers et al. 2017; Williams et al. 2022).

The use of genetically unmatched vaccines in Africa may contribute to the observed vaccine failures, as they could lead to suboptimal outcomes such as reduced efficacy in lowering viral shedding after a challenge. Consequently, there is a critical need to develop a new, more robust vaccine that can overcome these obstacles and deliver consistent protection. The present study addresses this pressing need by evaluating a new vaccine candidate designed to improve immunogenicity and stability, thus offering a viable solution for improving ND control across the continent. The present study evaluated the immunogenicity of two ND DNA-matched vaccines constructed from the F and HN genes of a virulent strain of NDV genotype VII isolated from Tanzania. One of the vaccines was adjuvanted with IL-28b. DNA vaccines offer several advantages for the eradication of NDV in Africa, particularly against virulent strains of the virus, such as genotype VII. DNA vaccines can be designed to contain multiple antigens, providing broader protection against different strains of NDV. This versatility is particularly important given the genetic diversity of NDV, which has multiple genotypes that cause different levels of disease in poultry populations globally (Zhao et al. 2014).

In the present study, we observed that pTwist-F-HN-VII-IL28b elicited a significant HI titer than pTwist-F-HN-VII and LaSota. Importantly, HI titers > log2^4^ or log2^5^ after vaccination are indicative of protective immunity against NDV (Kapczynski and King 2005). Additionally, an HI titer below log2^3^ is associated with diminished protection (OIE 2012). However, in this study, LaSota and pTwist-F-HN-VII, despite reaching an HI of log2^7^ two weeks after the booster, still exhibited a mortality rate of up to 40% during the challenge. The data obtained in this study indicate that the LaSota vaccine offers some clinical protection against current strains of NDV. Furthermore, the antigenic heterogeneity of circulating strains does not affect shedding patterns in vaccinated birds, as previously suggested by various authors (Dortmans et al. 2014; Naguib et al. 2021). This indicates that mortality rates of up to 100% should not be expected during outbreak periods, particularly on farms where chickens are vaccinated by their owners. It is therefore possible that some vaccine failures may be attributable to errors in the administration of the vaccine.

The lower mortality rate observed in the group vaccinated with the adjuvanted IL-28b vaccine (pTwist-F-HN-VII-IL28b) than in the LaSota- and pTwist-F-HN-VII-vaccinated groups suggested that the challenge virus replicated to a low extent and that the adjuvanted vaccine induced effective neutralizing immunity in these birds (Randall and Goodbourn 2008). As a member of the recently identified IFN-λ family, IL-28b (IFN-λ3) plays a vital role in adaptive immune responses to viral infections and, more specifically, in the antiviral defense of chickens. Using the IL-28b plasmid as a vaccine adjuvant enhances both humoral and cellular responses, highlighting its potential for future vaccine research (Morrow et al. 2010). IL-28b has the potential to be a useful adjuvant and can improve a vaccine’s capacity to generate virus-specific immune responses (Sabbaghi et al. 2021). Reuter et al. (2014) proposed the intriguing possibility that IL-28b may play a role in defending mucosal surfaces against viral intruders in most, if not all, vertebrates. The results of the two groups of chicks vaccinated with our novel vaccines in this study demonstrated that the differences observed in induced humoral immunity and survival to early challenge with virulent NDV can be influenced by the use of an adjuvant in vaccine development. The efficacy of pTwist-F-HN-VII-IL28b may be attributed to the presence of the adjuvant IL-28b, which may have induced and promoted a more robust protective immune response against NDV. It is well established that cytokines are immunostimulatory molecules (Ravikumar et al. 2022). These results support the conclusion that producing an inactivated, adjuvanted vaccine derived from local, circulating velogenic NDV is a productive method for protecting vaccinated birds from morbidity and mortality associated with velogenic strains (Fawzy et al. 2020). Another reason for turning to genotype-matched vaccines is that genotype-matched vaccines provide better protection by limiting viral shedding (Wajid et al. 2018). An experiment conducted by Absalón et al. (2019) with NDV genotype VII circulating in Latin America (Mexico, Colombia, Venezuela, and Peru) demonstrated enhanced outcomes, including a notable decline in the number of shedders and the level of virus shedding. This study provides an inaugural demonstration that incorporating IL28b as an adjuvant in an NDV vaccine enhances the protective efficacy of the vaccine when tested *in* chicks in vivo.

Although this will require many new trials and improvements in vaccine design, control and eradication of ND in Africa will be achieved through vaccination in the coming years, as has been done in other countries. In this context, one promising approach could be to study the immunogenicity stimulated by our pTwist-F-HN-VII-IL28b vaccine candidate by administering our vaccine to chickens using *in vivo jetPEI*, a cationic polymer that delivers DNA through electrostatic interactions with DNA and cell membranes. *In vivo jetPEI*^®^ (Polyplus Transfection, Illkirk, France) has already been successfully tested for its ability to deliver DNA into chicken embryos (Jordan et al. 2014). Additionally, for regulatory approval and licensing, robust data on vaccine performance are required (Hassanzadeh et al. 2024), and molecular confirmation of virus shedding needs to be performed in future studies with our genotype-adapted adjuvanted vaccine to confirm its efficacy in preventing virus replication and transmission for regulatory approval and practical field application.

## Conclusions

This study demonstrated that the LaSota vaccine, one of the most widely used vaccines on the African continent, remains an effective vaccine against NDV, although with some nuances, particularly in the presence of velogenic strains. The results of this study also demonstrated that the use of a DNA-adjuvanted genotype-matched vaccine could be considered one of most effective options for combating current velogenic NDV infection in the field in Tanzania and elsewhere in Africa. The efficacy and safety profile of pTwist-F-HN-VII-IL28b, as evidenced by our findings, suggest that it could play a pivotal role in reducing the prevalence and impact of ND across the continent. By improving flock health and boosting productivity, this vaccine has the potential to bring substantial economic benefits to poultry farmers and contribute to food security in the region. Further experimentation with a larger sample size is required to explore the comparative efficacy of this genotype-matched ND DNA adjuvanted and unmatched vaccines or their combinations, in the context of the challenges posed by the virulent strains of NDV in circulation.

## Data Availability

No datasets were generated or analysed during the current study.
